# Paroxysmal freezing of gait in a patient with mesial frontal transient ischemic attacks

**DOI:** 10.1186/s12883-017-0901-7

**Published:** 2017-06-28

**Authors:** Hee Won Hwang, Seung Ha Lee, Chul Hyoung Lyoo, Myung Sik Lee

**Affiliations:** Department of Neurology, Gangnam Severance Hospital, Yonsei University College of Medicine, Eonjuro 211, Gangnam-gu, Seoul, South Korea

**Keywords:** Mesial frontal lobe, Transient ischemic attack, Freezingof gait, Locomotor block

## Abstract

**Background:**

Rare patients have been reported who developed a mixture of gait disturbances following a focal lesion in the frontal lobe. Thus, the exact location of frontal lesion responsible for a specific gait disturbance is not well defined.

**Case presentation:**

We describe a 47-year-old man who experienced two episodes of paroxysmal freezing of gait of the right leg. During the attacks, he had no motor weakness, sensory change, or disequilibrium. He had past history of panic attacks. Recently, he had been under severe emotional stress. T2 and diffusion brain magnetic resonance imaging scans were normal. So far, the most likely clinical diagnosis might be functional freezing of gait. However, magnetic resonance angiography showed atherosclerosis in the proximal left anterior cerebral artery. Perfusion scans showed a delayed mean transit time in the left mesial frontal lobe. He developed two more attacks during the four months of follow up.

**Conclusions:**

The presented case illustrates that the mesial frontal lobe may be important in the pathophysiology of freezing of gait. We speculate that the supplementary motor area may generate a neuronal command for the initiation of locomotion that in our case may have been inhibited by a transient ischemia.

## Background

Freezing of gait (FOG) is defined as episodic brief absence or marked reduction of forward progression of the feet despite the intention to walk [1]. Patients with frontal gait disorder may have FOG. However, they frequently have widespread cerebrovascular lesions and present various combinations of additional gait disturbances including start and turn hesitation, short steps, shuffling, and postural instability. Thus, in patients with frontal gait disorder, exact location of brain lesion responsible for FOG is difficult to decipher [2].

Rare patients have been reported who developed FOG following a focal lesion involving the dorsal midbrain tegmentum, suggestive of important role of mesencephalic locomotor region in the locomotor initiation [3–5]. However, frontal lobe, especially the supplementary motor area, plays also a prominent role in the initiation and control of human locomotion [1]. Here, we describe a patient who developed paroxysmal FOG of the right leg associated with left mesial frontal transient ischemic attacks (TIAs).

## Case presentation

**Fig. 1:**
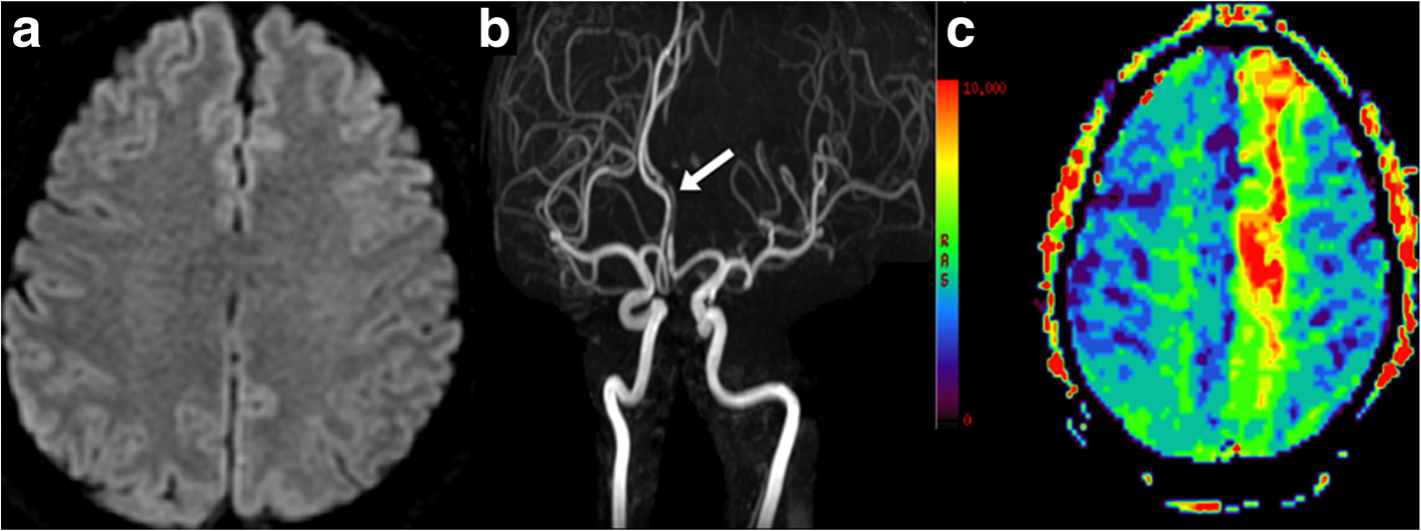
**a** Diffusion axial brain MRI scan shows no abnormalities. **b** Cerebral MR angiography shows atherosclerotic narrowing in the left anterior cerebral artery (*arrow*). **c** Perfusion axial brain MRI scan shows delayed mean transit time in the left mesial frontal lobe

A 47-year-old man visited the emergency room due to two incidents of transient locomotor blocks. He had a past history of panic attacks. Recently, he had been under severe emotional stress. When he attempted to walk at the pedestrian crossing on the green light, his right foot was glued to the ground. He sat down and massaged his right leg with both hands. Several seconds later, he could walk normally again. Two hours later, when he attempted to run while walking, his right foot was again stuck to the ground for several seconds. During the attacks, there were no knee trembling of the frozen leg. He denied any motor weakness, sensory change, or dyskinesia of the arms or legs. On neurological examination, he had no motor, sensory, or cerebellar dysfunctions. T2 and diffusion brain magnetic resonance imaging (MRI) scans were normal (Fig. 1a). However, magnetic resonance (MR) angiography showed atherosclerosis in the proximal left anterior cerebral artery (Fig. 1b). Perfusion scans showed a delayed mean transit time in the left mesial frontal lobe, including the supplementary motor area and cingulate cortex (Fig. 1c). Routine laboratory tests were all normal, including CBC, liver function tests, renal function tests, urine analysis, electrocardiography, and chest x-ray studies. There was no hyperlipidemia. Screening tests for autoimmune disease (e.g. ANA, Ani-dsDNA antibodies, P-ANCA, C-ANCA, Anti-Cardiolipin antibodies Ig M andIg G) were all negative. Transthoracic echocardiography study showed no abnormalities. Interictal electroencephalography (EEG) studies revealed no abnormalities. During four months of follow up period, he had been treated with low dose aspirin, but he developed two more attacks when he attempted walking.

## Conclusion and discussion

The presented case developed paroxysmal locomotor blocks. There were no motor weakness, sensory changes, or cerebellar ataxia. The attacks were induced by sudden movements of the leg as in paroxysmal kinesigenic dyskinesia, but there was no dyskinesia. Unlike the typical FOG observed in patients with degenerative parkinsonism, the present patient developed FOG in open spaces and there was no hastening or alternating knee trembling-like leg movements. Besides, he did not have any parkinsonian features [1]. T2 and diffusion weighted brain MRI studies showed no lesions (Fig. 1a). He had past history of panic attacks and recently had been under severe emotional stress. So far, the most likely clinical diagnosis might be a functional FOG [6].

However, cerebral MR angiography showed atherosclerosis in the left anterior cerebral artery (Fig. 1b) and perfusion MR scans showed ischemia in the left mesial frontal lobe (Fig. 1c). The presented case did not develop sensory dysfunctions or motor weakness during the attacks, probably because the lesion was remote from the sensory pathways and unilateral proximal limb receives motor inputs from the bilateral supplementary motor areas [7]. Indeed, about 20% of patients with anterior cerebral infarctions develop neither leg weakness nor sensory changes [8].

FOG occurs rarely as a form of bilateral akinetic seizure. Such FOG is precipitated by standing up, gait initiation, turning, stumbling, or startle induced by unexpected auditory or tactile stimuli [9]. Because mesial frontal epileptic discharge can hardly be detected by conventional EEG studies, epileptic origin of FOG can not be excluded in the presented case [10]. However, increased neuronal excitability in TIA causes dyskinesia (so call ‘shaking TIA’) rather than epileptic motor arrest [11]. FOG has been reported in patents who had widespread cerebral and bilateral basal ganglia lesions associated with hypoxia, carbon monoxide poisoning, wasp sting allergy, orpantothenate kinase associated neurodegeneration, as well as primary degenerative parkinsonism [12]. A recent neuronal network mapping study attributed FOG to the dorsal medial cerebellum [13]. FOG may also occur in patients with a focal brain lesion. The most often reported focal lesion is in the unilateral or bilateral dorsal midbrain tegmentum [3–5]. However, some patients had progressive worsening of gait disturbances and extensive subcortical white matter changes that obscured the causal relationship between the midbrain lesion and FOG [3].

We reviewed the literature and identified eight patients who developed gait disturbances or postural instabilities following a focal lesion in the frontal lobe. As the present case, seven of the eight patients had lesions in the mesial frontal lobe (Table 1) [14–21]. They developed various gait disturbances, inducing ataxic gait, shuffling gait, astasia, or FOG. At least four patients have been reported who developed FOG following a mesial frontal lesion associated with cortical vein thrombosis, lymphoma, or infarction [18–21]. However, they had additional neurological deficits that may disturb locomotor initiation (e.g. short steps, severe leg bradykinesia, spastic paraplegia, paratonia, retropulsion, loss of postural reflexes, and balance disturbance).

**Table 1:** Reported patients with gait disturbances or postural instabilities following a lesion confined to the frontal lobe

Reference	Lesion sites	Gait disturbances & postural instabilities	Other neurologic deficits	Characters of lesion
Sakakibara et al. [14]	bilateral medial frontal	shuffling gait	tinnitus, deafness, hallucination, versive seizure, disorientation, inattention	MRI: neurosarcoidosis
Chung et al. [19]	Lt. medial frontal	gait initiation disturbance	severe bilateral lower extremity bradykinesia	MRI: ischemic stroke
Nadeau et al. [20]	Bilateral parasaggital white matter	gait ignition difficulty	bilateral paratonia, retropulsion in sitting position	MRI: Primary CNS lymphoma
Ducruet et al. [15]	Rt. medial frontal	ataxic gait	Lt. hemiparesis	CT: hemorrhage
Wada et al. [16]	Rt. SMA	astasia		MRI: infarction
Robbins et al. [18]	Rt. Parasagittal frontal	freezing of gait	speech impairment, axial bradykinesia, balance disturbance	MRI: cortical vein thrombosis
Frassanito et al. [17]	Rt. basal frontal	gait instability		MRI: tumor excision
Della et al. [21]	Bilateral frontal	gait apraxia	spastic paraplegia, disequilibrium, loss of postural reflexes	MRI: infarction
Present case	Lt. mesial frontal	freezing of gait of the right leg		MRI: ischemia

*CT* computerized tomography, *MRI* magnetic resonance imaging, *SMA* supplementary motor area

The presented patient developed isolated paroxysmal FOG associated with mesial frontal TIAs. This finding indicates that the mesialfrontal lobe, probably the supplementary motor area, generates a neuronal command for the initiation of locomotion. The mesial frontal lobe lesion seemed to disrupt transmission of a locomotor initiation command from the mesial frontal cortex to the locomotor pattern generators in the subcortical (e.g. nucleus accumbens and ventral pallidum), brainstem (e.g. pedunculopontine nucleus, cuneiform nucleus, and subcuneiform nucleus),and spinal cord [1, 22].

## Abbreviations

ANA: Anti-nuclear antibodies
Anti-dsDNA: Anti-double stranded DNA antibodies
C-ANCA: Cytoplasmic anti-neutrophil cytoplasmic antibodies
CBC: Complete blood counts
FOG: Freezing of gait
MR: Magnetic resonance
MRI: Magnetic resonance imaging
P-ANCA: Perinuclear anti-neutrophil cytoplasmic antibodies
